# Preventable Social Ills

**Published:** 1913-02-01

**Authors:** 


### PREVENTABLE SOCIAL ILLS.

### II.—THE HABIT OF ILL-HEALTH.

Among the seven million children of school age in this country a proportion, varying according to locality, are doomed to grow up in the habit of ill-health. Medical inspection has detected them, has classified them as below average, has indicated the lines which treatment should take, and has passed on, recognising on the occasion of the next inspection that in the majority of cases the defects exposed have grown a little more salient, the vitality a little further impaired.

Medical inspection so far has chiefly benefited the children of parents able and willing to procure for them the requisite medical treatment. The number of cases which receive some form of treatment after inspection varies enormously according to the trouble taken by voluntary agencies and the facilities available. In Surrey it has been estimated that 28.3 of the cases in which treatment was recommended were subsequently attended to, and in Derbyshire the proportion rose to 63.3. Even the higher figure, however, cannot be considered satisfactory, especially in view of the fact that those who elude treatment are commonly the children who are exposed to the worst home conditions.

The Medical Treatment Act (1909) has compelled local educational authorities to make a charge for the treatment of school children, with power to remit payment in case of poverty. This has had the effect of restricting the extent to which parents are able to resort freely to the hospitals with their children of school age, and an impasse has been created, which has not been permanently removed by arrangements made between the London County Council and some hospitals. The physicians and surgeons in charge of out-patient departments have frequently exposed the inevitable defects of out-patient treatment for children. Not merely are the hospitals very unequally distributed, so that sick children are compelled to travel great distances to attend, but there is complete want of control as regards attendance and after-treatment, together with much overcrowding, and consequent difficulty in attending to all but the worst cases.

### Rise of the School Clinic.

The establishment of school clinics has been sanctioned by the Board of Education in various localities. The London County Council have one at Deptford, supplemented by a smaller centre at Bow, and aided by voluntary agencies. Another clinic has been formed at Blackfriars, in connection with St. George's Dispensary, and to this a dental clinic has been added as a private experiment. Bradford has a well-equipped school clinic. and so have Brighton, Southampton, York, and Reading. Cambridge has voluntarily started a dental clinic, which has proved so successful that it has been taken over by the municipal authorities. The school clinic at Wandsworth has been started under the auspices of the British Medical Association, and in no case has any friction been occasioned with local practitioners. But the school clinic is spreading very slowly.

Those who have read our articles dealing with its right organisation will hardly need reminding that in the first place, by this means, and by this alone, can the necessary treatment be secured for the child without delay and with a certainty of being properly administered. At present it is nobody's business to see that even the grosser defects observable in the health of scholars are rectified. By establishing a school clinic an authority is at once constituted whose business it is to take steps to heal the patients who now throng our schools untended. Every child who passes through the doctor's hands enters a new phase of hope in respect to the ailments from which he suffers, ailments which have in innumerable instances grown to seem natural and irremediable, and are in process of setting up the habit of ill-health which it is the business of the school doctor to break. Often the treatment required is daily or bi-weekly attention for months, even years, and regularity is the first condition of success. How different the continuity secured by school attendance and the nice attention to detail supplied by the school nurse, compared with the hasty visit to dispensary or sixpenny doctor paid by the ignorant mother, who misinterprets every direction, and constantly varies the treatment by trying another doctor or resorting to quack remedies. Again, often the main thing needed to restore health is a change of home conditions. Malnutrition may be the result of poverty or of ignorance, but in either case the school authority, which distributes free meals, is in a position to deal with it. The distinction between medicine and food grows very dim in certain slum districts, and many authorities believe that the school clinic is the best agency for selecting the children who need meals, and for arranging what form the meal should take. It is easy to see that many other forms of voluntary help, such as country holiday funds, boot and clothing clubs, etc., could be brought into partnership with the school clinic with great advantage to such charitable aids, in which overlapping is a too frequent feature. It has been surmised that in one or two thousand cases the Board of Guardians and the local education authorities are simultaneously feeding the same children. Such abuses would disappear if free meals were provided as a regular part of the treatment of necessitous and ailing children at the school clinic. The discovery in any school of a continuous stream of children all presenting the same defects traceable to the same bad habits would indicate the need for such instruction in personal hygiene as is already given with the best results in the "defective" schools. By aid of the school nurses the standard of the home could be raised, and the work of the teachers facilitated by the removal of the most serious obstacle to good education. The provision of baths, of open-air schools, and of gymnasia would probably follow the school clinic in many places. And the reasons for including in it a dental department are too strong to be resisted.

We have said that the habit of ill-health starts usually in childhood. It is largely due to inherited defects, to extreme poverty, to the vice or ignorance or neglect of parents. The invalid child is the saddest blot among the many blots in our social system. But the ray of hope which illumines the group of sick children treated in the school clinic with simple remedies, fresh air, food, and cleanliness is found in the swift responsiveness of young lives to the good influences set stirring around them. These child-minds are extraordinarily sensitive to impressions of comfort, kindness, and good cheer. Mr. Haden Guest has observed:* "A laughing and a smiling child should be the rule, a solemn or a weeping child the exception." Nature works for the child's health, and in no particular more efficaciously than when she sets stirring in him that buoyant unconsciousness of bodily functions which we recognise as the habit of health. When this has once been lost it would seem that nothing short of instituting a discipline of health as part of the regular school routine will suffice to restore it.

* *The Case for School Clinics*, by L. Haden Guest, M.R.C.S., L.R.C.P.

